# PAI-1: An Integrator of Cell Signaling and Migration

**DOI:** 10.1155/2011/562481

**Published:** 2011-08-03

**Authors:** Ralf-Peter Czekay, Cynthia E. Wilkins-Port, Stephen P. Higgins, Jennifer Freytag, Jessica M. Overstreet, R. Matthew Klein, Craig E. Higgins, Rohan Samarakoon, Paul J. Higgins

**Affiliations:** Center for Cell Biology and Cancer Research, Albany Medical College, 47 New Scotland Avenue, Albany, NY 12208, USA

## Abstract

Cellular migration, over simple surfaces or through complex stromal barriers, requires coordination between detachment/re-adhesion cycles, involving structural components of the extracellular matrix and their surface-binding elements (integrins), and the precise regulation of the pericellular proteolytic microenvironment. It is now apparent that several proteases and protease inhibitors, most notably urokinase plasminogen activator (uPA) and plasminogen activator inhibitor type-1 (PAI-1), also interact with several cell surface receptors transducing intracellular signals that significantly affect both motile and proliferative programs. These events appear distinct from the original function of uPA/PAI-1 as modulators of the plasmin-based proteolytic cascade. The multifaceted interactions of PAI-1 with specific matrix components (i.e., vitronectin), the low-density lipoprotein receptor-related protein-1 (LRP1), and the uPA/uPA receptor complex have dramatic consequences on the migratory phenotype and may underlie the pathophysiologic sequalae of PAI-1 deficiency and overexpression. This paper focuses on the increasingly intricate role of PAI-1 as a major mechanistic determinant of the cellular migratory phenotype.

## 1. Introduction

**Figure 1:**
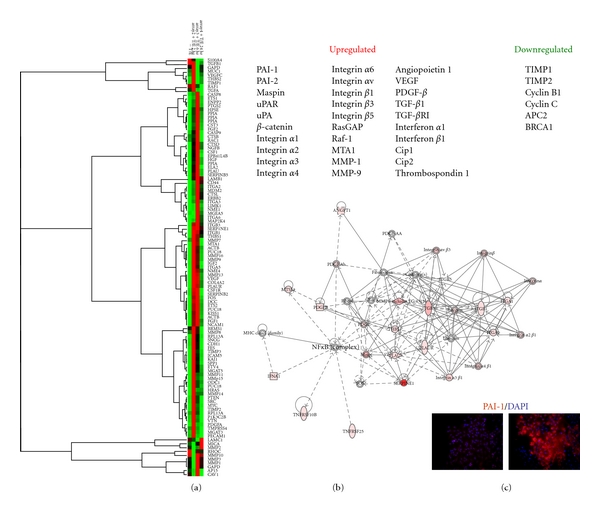
Transcriptome profiling and pathway analysis of the cellular plastic response upon combined exposure to transforming growth factor-*β*1 (TGF-*β*1) and epidermal growth factor (EGF). Microarray heat map of dual growth factor-stimulated HaCaT II-4 human keratinocytes illustrating the increased expression of mRNAs encoding proteins involved in the control of pericellular proteolysis, migration, and stromal invasion (a). PAI-1 transcripts were the most highly upregulated (170-fold), induced early (with 6 hours) after addition of TGF-*β*1+EGF and prior to acquisition of the migratory phenotype. The Ingenuity pathway clustergram illustrates potential functional interactions among the repertoire of induced genes (b). Pathway analysis of many of the affected genes (Table) indicate that several including uPA, uPAR, SERPINE1 (PAI-1), and MMPs are TGF-*β*1 targets and encode critical elements in the integrative proteolytic cascades that regulate matrix remodeling and stromal invasion. Immunocytochemistry of paraformaldehyde-fixed, detergent-permeabilized, HaCaT II-4 cells that were serum-starved then stimulated with TGF-*β*1+EGF for 5 hours indicated that increased PAI-1 mRNA abundance reflected an early up-regulation in immunocytochemically-detected PAI-1 protein (c). Left panel: unstimulated cells, right panel: TGF-*β*1+EGF-stimulated keratinocytes. Nuclei were visualized with DAPI. © 2000–2009 Ingenuity Systems. Inc. All rights reserved.

**Figure 2:**
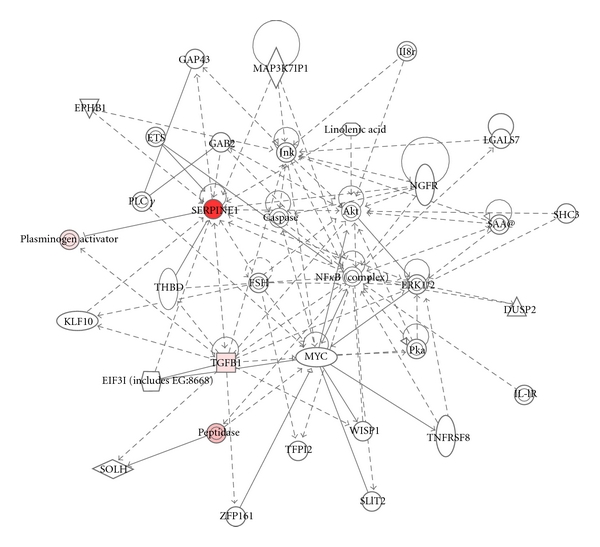
Integration of PAI-1 into cellular motile/proliferative pathways. Clustergram analysis of microarray data positions PAI-1 as a hub element both as a target and initiator (or inhibitor) of various pathways that regulate cellular motile (e.g., uPA, TGF-*β*1), proliferative (e.g., ETS, MYC, AKT), and survival/stress (e.g., JNK, caspase, NF*κ*B, TNFR) programs.

**Figure 3:**
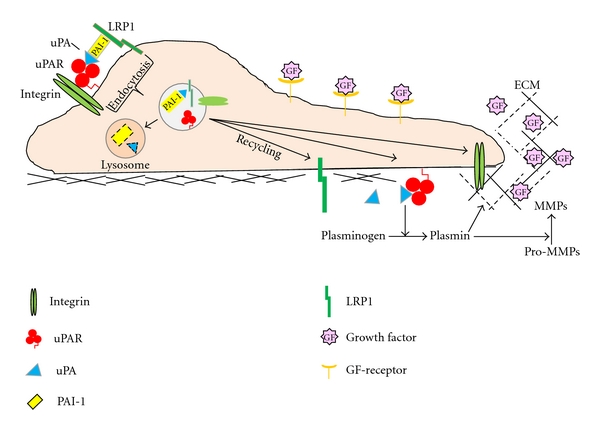
PAI-1 modulates cell migration by regulating ECM proteolysis. Physiological control of pericellular proteolysis occurs primarily through the regulation of plasminogen activation at the cell surface, which, in turn contributes to downstream MMP activity. Focal proteolysis disrupts ECM architecture, breaking cell-matrix interactions with receptors, such as integrins, and releasing bioactive fragments of extracellular matrix molecules, as well as growth factors that stimulate migratory behavior. PAI-1, through its ability to inhibit uPA-dependent activation of plasmin, titers this process maintaining the scaffolding necessary to facilitate cell migration. PAI-1: plasminogen activator inhibitor type-1, uPA: urokinase-type plasminogen activator, uPAR: uPA receptor, MMP: matrix metalloproteinase, GF: growth factor, LRP1: low-density lipoprotein receptor-related protein-1.

The switch between a sessile and migratory cellular phenotype is triggered, in part, by the activation of signaling pathways that regulate the expression of the involved genes, (e.g., [1, 2]). While the actual genomic response varies as a consequence of cell type, the acquisition of a core “plasticity” signature (at both the mRNA and proteomic levels) represents the transition to a motile phenotype whether over simple planar surfaces or through complex matrix barriers in normal as well as transformed keratinocytes, (e.g., [2–7]). Global transcriptome profiling of both wounded keratinocyte cultures and epithelial tumor cells has highlighted the requirement for precise spatial/temporal control of pericellular proteolysis and matrix remodeling in the integration of the cellular motile/tissue repair responses [2, 5]. Indeed, among the transcriptional outputs (i.e., genes with altered expression) that typify the migratory or invasive phenotype, urokinase plasminogen activator (uPA) and its major negative regulator plasminogen activator inhibitor type-1 (PAI-1) are among the most highly induced transcripts, (e.g., [4, 5, 8]) (Figure 1). PAI-1 belongs to the **ser**ine **p**rotease **in**hibitor (SERPIN) protein family that also includes PAI-2 and PAI-3 (protein C inhibitor), protease nexin-1, and neuroserpin (reviewed in [9]). uPA and PAI-1 (also known as SERPINE1) are both the targets and modifiers of pathways that impact proliferative/migratory events (Figure 2) and coordinately titrate the overall pericellular proteolytic balance directly (via generation of plasmin) as well as indirectly by activating several members of the matrix metalloproteinase (MMP) family (reviewed in [4, 7]). Motile epithelial cells focalize both uPA, following interaction with its cell surface receptor uPAR, and PAI-1, upon binding of this SERPIN to uPA/uPAR or vitronectin (VN), to the leading edge where they modulate the interrelated events of matrix remodeling and migration, (e.g., [10–12]). Focal proteolysis reorganizes extracellular matrix (ECM) architecture, affecting cell-ECM interactions with integrin receptors and releasing bioactive fragments of matrix molecules as well as activating growth factors that stimulate the migratory behavior (Figure 3) (reviewed in [7]). These findings have important implications. While uPA and uPAR are widely implicated in tumor invasion, deficiencies in PAI-1 levels also correlate with significantly reduced epithelial cell migration and tumor progression [1, 4, 7, 13]. A critical balance between uPA and PAI-1 appears required, therefore, to create a microenvironment compatible with efficient cell motility. High stromal PAI-1 levels, in fact, correlate with a poor prognosis in various cancers [14–16] and typify diseases in which fibrosis and/or cellular infiltration are common pathologic features (e.g., scarring anomalies, renal fibrosis, atherosclerosis) [17–21]. Collectively, these findings suggest that PAI-1-dependent preservation of the surrounding matrix may facilitate cell locomotion *in vivo*, perhaps by fine-tuning the proteolytic activity to optimize tissue penetration. This paper focuses on the most recent developments in this field and on the complex proteolytic as well as nonproteolytic functions of PAI-1 in the cellular motile program. 

## 2. PAI-1-Regulated Cell Migration: Receptor Interactions

**Figure 4:**
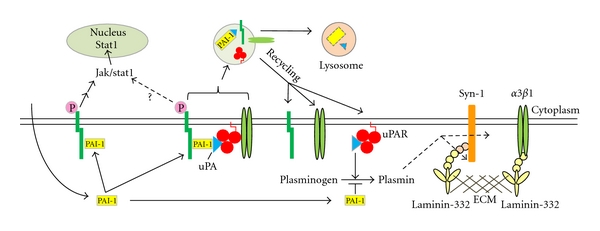
PAI-1 modulates migration through cell surface receptors. PAI-1 binding to LRP1, in a non-uPA/uPAR-dependent manner, triggers Jak/Stat1 signaling events that culminate in enhanced cell migration (left). It is unclear whether this process requires PAI-1 interaction with the ECM. PAI-1 binding to uPA/uPAR results in the internalization of the PAI-1/uPA/uPAR complexes in an LRP1-dependent manner (middle). PAI-1 binding to uPA/uPAR can also trigger the detachment of cell surface integrins from their ECM ligands and subsequent internalization in an LRP1-uPA/uPAR-dependent manner. In each case, receptors (integrin, uPAR, LRP1) recycled back to the cell surface, while uPA and PAI-1 are degraded. PAI-1, through its ability to titer active plasmin, may also promote syndecan-1-dependent migration on unprocessed laminin-332 by preventing the cleavage of the syndecan-binding site LG4/5 (right). Additionally, inhibition of plasmin activation by PAI-1 facilitates migration on unprocessed laminin-332 by reducing the shedding of syndecan-1 from the cell surface. As the proteolytic environment matures and PAI-1 levels decrease, integrins *α*3*β*1 and *α*6*β*4 (not shown) engage the proteolytically-cleaved or processed form of laminin-332 facilitating construction of hemidesmosomes.

 Stromal PAI-1 is itself a substrate for several extracellular proteases including elastase, MMP-3, and plasmin [22–24]. “Cleaved” PAI-1 is unable to interact with its target plasminogen activators uPA and tissue-type PA (tPA) to inhibit plasmin-based proteolysis but retains its ability to bind the low-density lipoprotein receptor-related protein-1 (LRP1) and augment cell migration, through a u/tPA complex-independent interaction (Figure 4, left) [25]. LRP1, in addition to its function as a major endocytic receptor for multiple ligands, is also a key signaling mediator in several pathways due, in part, to its ability to support interactions with multiple adaptor and scaffolding proteins [26]. LRP1 ligand binding and/or its complex formation with cell surface partners including integrins [27–29], growth factor receptors [30–32], and proteoglycans [33] activates mitogen-activated protein (MAP) and nonreceptor *src* kinases [34–37], impacting cell proliferation [30, 31, 38, 39] and migration [25, 34, 40] with the motile response involving activation of Rho family GTPases [40]. Alternatively, PAI-1 can also function as a signaling molecule that directly affects cell migration through engagement of LRP1 and the very low-density lipoprotein receptor [41]. Indeed, the different conformations of PAI-1 (active, latent, cleaved) interact with LRP1 to stimulate cellular migration into 3D collagen gels through a LRP1-dependent mechanism [42]. All three forms of PAI-1 increase LRP1-dependent cell motility with the activation of the Jak/Stat1 pathway [25, 43, 44] (Figure 4, left). While active PAI-1 is routinely cleared from the extracellular environment in a complex with uPA/uPAR/LRP1, latent and cleaved species of PAI-1, with a preserved motile function, remain embedded in the matrix likely serving as a reservoir to maintain cell movement. 

One prerequisite for efficient cellular migration is a sustainable, flexible state of cell adhesion. PAI-1 significantly impacts adhesion through interaction with LRP1 and VN. PAI-1 mutants that vary in their capacity to bind uPA, VN, or LRP1 can attenuate smooth muscle cell adhesive forces through deregulation of integrin activity [27]. This mechanism, targeting only active, matrix-engaged integrins, results in cell detachment from VN, fibronectin (FN), and collagen matrices [45], allowing for readhesion to alternative matrix structural elements, thus promoting migration. It appears that even low concentrations of PAI-1 lead to substantial and rapid changes in the actin cytoskeleton and the loss of focal adhesions [25] with likely consequences on the motile phenotype. 

PAI-1 also regulates levels of cell surface integrins by triggering their internalization in an LRP1-dependent manner [27, 45, 46] resulting in cell detachment from various substrates [27, 45] (Figure 4, middle). Integrin internalization by LRP1, however, is not a requirement during PAI-1-initiated cell release [45]. This mechanism appears to differ from that which modulates PAI-1-stimulated migration directly via LRP1, as uPA and uPAR are required for deadhesion but not for the migratory response [25, 27, 43, 46]. Although LRP1-mediated integrin endocytosis seems not to be necessary for efficient cell detachment, integrin endocytosis would allow for their subcellular redistribution (i.e., to the leading edge) in support of cell locomotion and stromal invasion. While the interaction between PAI-1 and uPA/uPAR/integrin complexes would ultimately enhance the integrin/uPAR “*attachment-detachment-reattachment*” cycle [47], thereby, increasing cell motility, it is apparent that PAI-1 can utilize multiple avenues to impact LRP1-dependent cell migration (Figure 4, left and middle). Further complicating this process is the potential for PAI-1 to modulate syndecan-dependent keratinocyte migration, as evident during wound healing. Keratinocytes at the wound margin begin to synthesize and deposit unprocessed laminin-332, supporting syndecan-1 binding through the LG4/5 domain (Figure 4, right). PAI-1, which is also expressed by cells at the wound edge, stabilizes this interaction by preventing plasmin-initiated proteolytic processing of laminin-332 [48] and syndecan-1 shedding [49, 50]. The presence of VN at the wound edge can augment this event through its ability to focalize PAI-1 and extend the half-life of active PAI-1 (discussed below) as well as engage syndecan-1 [51]. PAI-1, through its ability to reduce pericellular levels of active plasmin, promotes syndecan-1-dependent migration on unprocessed laminin-332 by preventing cleavage of the syndecan-binding site LG4/5. Additionally, PAI-1 inhibition of plasmin activation facilitates migration on unprocessed laminin-332 by reducing the shedding of syndecan-1 from the cell surface. As the proteolytic environment matures, PAI-1 and VN are endocytosed and degraded [52, 53]. Syndecan-1 binding is lost due to proteolytic processing of laminin-332, as well as syndecan-1 ectodomain shedding; *α*3*β*1 binding to processed laminin-332 begins to slow keratinocyte migration and initiate hemidesmosome formation [48, 54] (see Figure 4, right).

## 3. PAI-1-Regulated Cell Migration: Interactions with Vitronectin

PAI-1/VN interactions impact several mechanisms associated with cell migration. Whereas PAI-1 had been recognized earlier as a highly significant prognostic indicator for malignant disease outcome [55], the importance of stromal VN as inducer of cell motility came in focus only more recently [56–58]. In part, it does so by stabilizing PAI-1 in an active conformation, extending its half-life and amplifying the inhibition of focal proteolysis modulating the extent, locale, and duration of matrix remodeling, thereby preserving a stromal architecture permissive for cell motility [59, 60]. This is particularly important following cutaneous injury where restoration of barrier function and tissue integrity is dependent upon keratinocyte movement. PAI-1 and VN are both released from the *α* granules of platelets during hemostasis, where their combined presence would presumably promote the formation of a fibrin clot and subsequently contribute to provisional matrix remodeling [61, 62]. PAI-1 upregulation in keratinocytes at the wound margin [1, 12] highlights the potential involvement of this SERPIN in initiating tissue repair. VN expression, however, is limited under normal physiological conditions [63–66] but similarly enhanced under circumstances requiring stromal remodeling (i.e., wound repair [67–69] or tumor progression [70–74]) suggesting a continuing, albeit dynamic, molecular interaction with PAI-1 of potential physiologic significance. This dynamic might reflect the fact that the binding of PAI-1 to VN alters the motogenic properties of PAI-1, rendering PAI-1/VN complexes nonmotogenic, whereas all non-VN-bound PAI-1s (cleaved, latent, or active) exhibit strong motogenic properties [43]. 

**Figure 5:**
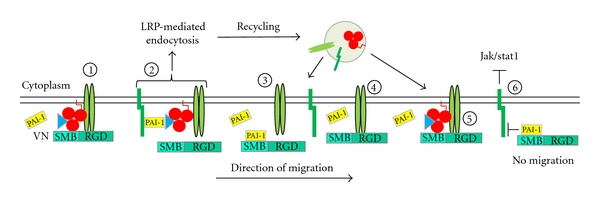
PAI-1-VN interactions disrupt the receptor binding events and regulate cell adhesion. On a VN matrix, cells are attached through binding of uPAR and *α*_v_ integrins to VN, and these interactions are supported by uPA in complex with uPAR (step 1). Secreted PAI-1 will bind to and inactivate uPA, consequently decrease the affinities of uPAR and integrins for VN, and initiate cell detachment and subsequent LRP1-mediated endocytic clearance of those quaternary complexes (step 2). Excess extracellular PAI-1 can now bind to the unoccupied SMB domain in VN and prevent reattachment of uPAR to that site as well as *α*_v_ integrins to the adjacent RGD sequence (step 3). Once recycled integrins are engaging with unoccupied VN, PAI-1 is unable to displace these integrins competitively (step 4). Those *α*_v_ integrins are then available for complex formation with recycled uPAR in the presence of uPA (step 5; see also step 1). In a similar manner, VN binding to PAI-1 inhibits the interaction of PAI-1 with LRP1 and, as a consequence, prevents Jak/Stat1-mediated migration (step 6). Collectively, this may promote cell movement away from a PAI-1-abundant VN-rich matrix onto an alternative substrate that cannot be saturated by PAI-1 and where PAI-1 only regulates cell attachment through interaction with uPA/uPAR complexes.

The interaction between PAI-1 and VN also affects cell motility through mechanisms that directly modulate cell surface receptor binding (Figure 5). VN promotes cellular locomotion via RGD-dependent interactions with *α*v*β*3 and *α*v*β*5 integrins [75–78], as well as through binding to uPAR [79, 80]. The recognition site for PAI-1 on VN, however, approximates those for both integrin and uPAR docking [81], and, as a result, the interaction of PAI-1 with VN regulates the ability of these receptors to engage VN [47, 79–82] (Figure 5). PAI-1, in addition to regulating cell-to-substrate attachment, also affects cellular release from VN by two distinct mechanisms. The affinity of PAI-1 for VN is significantly higher than that of uPAR for VN. Consequently, PAI-1 can competitively displace uPAR from VN, initiating detachment of cells that rely mainly on uPAR for cell adhesion to VN [79, 80, 82]. However, PAI-1 is unable to promote its binding to VN by competitive displacement of preengaged integrins from VN. In the presence of uPA/uPAR/*α*v-integrin complexes; moreover, PAI-1 binding to complexed uPA will initiate integrin deactivation, promoting their detachment from VN and endocytic clearance [27, 45]. These receptors are subsequently recycled back to the cell surface to reengage matrix molecules and promote cell migration [26] (Figure 4, middle). In contrast to the effects of PAI-1 on cell attachment, the deadhesive effect of PAI-1 is strictly uPA-dependent and VN-independent since PAI-1 can also initiate cell release from FN, collagen-I, and laminin-332 matrices [45]. 

In addition, PAI-1/VN binding blocks PAI-1 interaction with LRP1, thus preventing the LRP1-dependent migration signaling [43] (Figure 5). The question remains how PAI-1 will react to the presence of the other two binding partners, VN and uPA. Recent observations would suggest that the stoichiometry between these three molecules will determine the result of their interactions [41]. Migration of human vascular smooth muscle cells on 2D and through 3D collagen gels, in the presence of VN, was significantly reduced in low PAI-1, whereas high PAI-1 concentrations strongly promoted cell migration.

## 4. Summary

Cell migration requires the temporal/spatial regulation of a series of complex proteolytic events coupled with the activation of critical surface receptors (uPAR, integrins, LRP1) and initiation of downstream signaling, by several elements intimately involved in pericellular proteolysis. PAI-1, through its varied interactions with VN and cellular receptors, is centrally positioned to coordinate the duration and locale of both intracellular (signal initiation) and extracellular (detachment/readhesion cycles, receptor binding) events that manage the intricate process of cell movement in both physiologic and pathologic contexts. Clearly, the binding of PAI-1 with its several targets including VN, uPA, uPA/uPAR, and LRP1 has the potential to affect the motile program on multiple levels providing opportunities to therapeutically manipulate this pathway in pathophysiologic settings.
